# Metabolic syndrome is associated with more pain in hand osteoarthritis: Results from the DIGICOD cohort

**DOI:** 10.1016/j.ocarto.2025.100573

**Published:** 2025-02-05

**Authors:** Alix Charton, Romane Lacoste-Badie, Sophie Tuffet, Alexandra Rousseau, Emmanuel Maheu, Bruno Fautrel, Maxime Dougados, Francis Berenbaum, Alice Courties, Jérémie Sellam

**Affiliations:** a Assistance Publique – Hôpitaux de Paris (APHP), Sorbonne University, Rheumatology Department, Saint-Antoine Hospital, Paris, France; b AP-HP, Sorbonne University, Clinical Pharmacology Department and Clinical Research Platform of Eastern Paris (URCEST, CRB, CRC), Saint-Antoine Hospital, Paris, France; c AP-HP, Sorbonne University, Rheumatology Department, La Pitié Salpêtrière Hospital, Paris, France; d AP-HP, University of Paris, Rheumatology Department, Cochin Hospital, Paris, France; e Centre de Recherche Saint-Antoine (CRSA), Inserm UMRS_938, Paris, France

**Keywords:** Metabolic syndrome, Hand osteoarthritis, Pain, Patient-reported outcomes

## Abstract

**Background:**

The role of metabolic syndrome (MetS) in osteoarthritis (OA) pain, particularly in non-weight-bearing joints like the hand (HOA), remains debated. This study assessed whether MetS is linked to increased hand pain in patients with HOA.

**Methods:**

Using the DIGICOD cohort, 352 HOA patients (85 ​% women, mean age 66.4 ​± ​7.4 years) were analyzed. Pain levels were evaluated via visual analog scale (VAS), AUSCAN pain subscore, and AIMS2 pain subscore. The presence of MetS (Adult Treatment Panel III criteria) and its components were assessed alongside demographic and clinical characteristics, including BMI and radiological severity (KL sum score). Associations were adjusted for confounders (age, sex, KL score, and HAD scale). Outcomes were dichotomized into high/low pain levels, with results expressed as odds ratios (OR) and 95 ​% confidence intervals (CI).

**Results:**

MetS was present in 36 ​% of patients and associated with higher pain levels during activity (VAS OR ​= ​1.61, 95 ​% CI 1.02–2.57) and overall OA pain (AIMS2 OR ​= ​1.85, 95 ​% CI 1.14–2.99). Adjusted AUSCAN pain subscore also correlated with MetS (OR ​= ​1.66, 95 ​% CI 1.05–2.62), but significance was reduced when adjusting for HAD (OR ​= ​1.56, 95 ​% CI 0.98–2.48). Elevated triglycerides, a MetS component, were significantly linked to higher AIMS2 pain scores (OR ​= ​2.58, 95 ​% CI 1.09–6.07). BMI was not found to be independently associated with pain.

**Conclusion:**

MetS correlates with increased pain in HOA, independent of structural damage and anxiety/depression, underscoring its systemic impact on OA-related pain.

## 1 Introduction

Hand osteoarthritis (HOA) is characterized by multiple symptoms including hand pain, stiffness, functional impairment, decreased grip strength, esthetic discomfort, and compromised quality of life [1,2]. HOA is often associated with other anatomical sites, particularly the knee and the hip, and can belong to generalized OA [3,4]. Currently, no curative treatment exists for OA, with therapeutic interventions primarily focusing on symptom management, especially pain relief. Thus, a better understanding of OA-related pain in HOA is critical to optimize the therapeutic management of these patients.

Pain in HOA, as in other sites, is a multifaceted phenomenon involving various mechanisms, with nociceptive pain involving bone remodeling and synovitis [5]. Moreover, pain may also originate from central sensitization, particularly in chronic OA, causing widespread or diffuse pain beyond the primary joint site [6,7]. Local inflammation plays a central role in the pathophysiology of pain, mediated by the release of a wide range of inflammatory mediators, including cytokines, adipokines, lipids and nitric oxide, which may originate from adipose tissue [5].

Furthermore, the OA-related pain can significantly differ among individuals, with patient-reported outcome measures (PROMs) serving as crucial tools to capture this diversity. Notably, while the visual analog scale (VAS) offers a quantifiable measure of pain intensity according to context, questionnaires like the Australian/Canadian Osteoarthritis Hand Index (AUSCAN) [8] provide a composite assessment of symptoms, including pain including pain in various situations. Consequently, not all PROMs contain the entirety of the OA-related pain experience.

Beyond joint-specific pathology and pain sensitization, systemic factors such as gender or psychosocial comorbidities (e.g. depressed mood) contribute to OA pain. Along this line, metabolic syndrome (MetS) is emerging as a potential systemic factor influencing OA-related pain, due to the role of low-grade systemic metabolic inflammation, the metainflammation.

MetS, characterized by the co-occurrence of several cardiovascular risk factors, has diagnosis criteria established by the Adult Treatment Panel III (supplemental file, Table 1), including elevated waist circumference, triglyceride levels, reduced HDL cholesterol, high blood pressure, and elevated fasting glycemia [9].

**Table 1:** Description of the study population and comparison between MetS+ and MetS- (n ​= ​352).

Variable	Total population N ​= ​352		Metabolic syndrome N ​= ​125		No metabolic syndrome N ​= ​227		p-value
	n (%) or m ​± ​sd ou P50 [P25; P75]						
	**N**^a^		**n**^a^		**n**^a^		
Sex	352		125		227		0.38^b^
Female		298 (84.7)		103 (82.4)		195 (85.9)	
Age at inclusion (years)	352	66.4 ​± ​7.4	125	67.9 ​± ​7.2	227	65.6 ​± ​7.3	0.005^d^
Body Mass index	347	25.1 ​± ​4.3	125	27.1 ​± ​4.5	222	24.0 ​± ​3.8	<0.0001^d^
BMI (WHO classification)	347		125		222		<0.0001^c^
Underweight (<18.5)		9 (2.6)		1 (0.8)		8 (3.6)	
Normal body weight (18.5–24.9)		183 (52.7)		45 (36.0)		138 (62.2)	
Overwheight (25–29.9)		116 (33.4)		48 (38.4)		68 (30.6)	
Obese (≥30)		39 (11.2)		31 (24.8)		8 (3.6)	
Metabolic syndrome components							
High waist circumference	352	102 (29.0)	125	64 (51.2)	227	38 (16.7)	<0.0001^b^
High triglycerides	352	133 (37.8)	125	117 (93.6)	227	16 (7.0)	<0.0001^b^
Low HDL cholesterol	352	123 (34.9)	125	111 (88.8)	227	12 (5.3)	<0.0001^b^
High blood pressure	352	267 (75.9)	125	115 (92.0)	227	152 (67.0)	<0.0001^b^
Elevated fasting blood glucose	352	87 (24.7)	125	53 (42.4)	227	34 (15.0)	<0.0001^b^
Sum of the Kellgren Lawrence sum score (0–128)	352	46.1 ​± ​17.6	125	48.6 ​± ​17.6	227	44.8 ​± ​17.4	0.05^d^
HAD scale - total score (0-21)	352	12.7 ​± ​6.1	125	13.8 ​± ​6.7	227	12.0 ​± ​5.7	0.01^d^
Current tobacco use	348	26 (7.5)	122	12 (9.8)	226	14 (6.2)	0.22^b^
Ongoing Paracetamol treatment	352	112 (32.2)	123	44 (35.8)	225	68 (30.2)	0.29^b^
Ongoing Oral NSAID treatment	348	61 (17.5)	123	22 (17.9)	225	39 (17.3)	0.90^b^
Ongoing grade II Painkiller	348	28 (8.0)	123	13 (10.6)	225	15 (6.7)	0.20^b^

BMI = Body Mass Index.

WHO = World Health Organization.

HOA = Hand Osteoarthritis.

NSAID = Systemic non-steroidal anti-inflammatory drugs.

HAD = Hospital Anxiety and Depression.

Wilcoxon-Mann-Whitney test.

a Number of data available.

b Chi-square test.

c Fisher's exact test.

d Student's *t*-test.

The relationship between MetS and the risk of OA disease has been debated, with conflicting results, depending on the adjustment factors and the joint studied [6,[10], [11], [12], [13]]. Notably, in the knee, the association between MetS and OA remains controversial, because of the strong correlation between MetS and obesity, a major risk factor for knee OA [8,[11], [12], [13], [14]]. However, analyzing HOA provides a relevant approach for studying the association between MetS and OA pain to avoid the strong influence of weight [6,13] and potentially to highlight the systemic role of MetS and of metainflammation in OA symptomatology, as previously demonstrated in HOA disease risk [[15], [16], [17]]. To date, one study indicated an association between MetS and painful interphalangeal OA after adjusting for age and BMI but without exploring association with pain intensity [18,19].

Herein, our main objective was to determine whether the presence of MetS in HOA patients is associated with increased pain. Additionally, we explored the potential association between each MetS components and OA-related symptoms and possible association between BMI and outcomes.

## 2 Methods

### 2.1 Study design and participants

This cross-sectional study involved patients enrolled in the hospital-based Digital Cohort Design (DIGICOD) cohort for HOA, with complete baseline data availability (NCT01831570). DIGICOD is a single-center, university hospital-based prospective cohort comprising patients with symptomatic HOA, irrespective of pain intensity threshold [20]. The study obtained approval from the appropriate ethics committee and adhered to current Good Clinical Practices guidelines. Patients were recruited between April 2013 and June 2017 and underwent annual assessments for six years, including clinical evaluations, imaging (hand and other symptomatic joint X-rays), and biological sampling every three years. Hand radiographs were evaluated using Kellgren-Lawrence (KL) [21].

Inclusion criteria comprised age >35 years and symptomatic HOA fulfilling American College of Rheumatology criteria [22], with ≥2 symptomatic joints among proximal/distal interphalangeal joints or KL ​≥ ​2 in the first interphalangeal joint or symptomatic thumb base OA with KL ​≥ ​2. The KL sum score represents the total of Kellgren-Lawrence (KL) grades assigned to all interphalangeal and metacarpophalangeal joints of both hands, with each joint scored from 0 (no OA) to 4 (severe OA), resulting in a total score ranging from 0 to 128. This metric reflects the overall extent of radiographic OA damage in the hands. Exclusion criteria included inflammatory destructive arthritis, gout/chondrocalcinosis, tophaceous gouty arthritis, secondary HOA, or rare genetic OA.

MetS was defined according to the ATP III criteria (see Supplementary File, Table 1), with MetS considered present if the patient met at least three of the specified criteria: elevated waist circumference (≥102 ​cm in men, ≥88 ​cm in women); elevated triglycerides (≥1.5 ​g/L or drug treatment for high triglycerides); reduced HDL cholesterol (<0.4 ​mg/dL in men, <0.5 ​mg/L in women or drug treatment for reduced HDL cholesterol); hypertension (systolic blood pressure ≥130 ​mm Hg, diastolic blood pressure ≥85 ​mm Hg, or antihypertensive medication); and elevated fasting blood glucose (≥5.6 ​mmol/L or blood glucose-lowering medication). Each component was assessed based on data collected at the initial (V0) visit. We searched for an association between MetS and pain in HOA using the composite questionnaire AUSCAN VA3.0 [8], moreover we used other several outcomes measuring different aspects of OA-related pain: VAS for hand pain at rest (0–100 ​mm), VAS hand pain during activity (0–100 ​mm), the short form of the AIMS-2 questionnaire focusing on OA in general and not targeting specifically one joint (ie not limited to the hands) [23], and the number of tender hand joint at pressure.

Each AUSCAN subscores was normalized on a 0–100 scale, as well as the FIHOA score.

### 2.2 Statistical analysis

The population studied consists of patients from the DIGICOD cohort for whom all components of MetS, pain outcomes and adjusting factors were available.

The patient characteristics were described in the entire population as well as according to the presence or absence of MetS. Qualitative variables are described by their frequency and number and compared between the two groups by a Chi-square test or a Fisher exact test if necessary. Quantitative variables following a normal distribution are described by their mean and standard deviation, the means of the two groups are compared by a Student's t-test. Quantitative variables that do not follow a normal distribution are described by the median and interquartile range and the distributions of the two groups are compared by a Wilcoxon-Mann-Whitney test.

All pain, function and stiffness outcomes were treated as quantitative variables and were binarized using the median values of the whole DIGICOD Cohort, which defined high and low symptom levels.

Then, to search for an association between MetS and symptoms, we performed an unadjusted analysis and two adjusted analysis using logistic regression models:

- Model 1: adjusted for age, sex, and the radiological KL total sum score of both hands
- Model 2: model 1 ​+ ​the Hospital Anxiety and Depression Scale (HAD) [24].

The selection of these adjustment factors was guided by well-established literature on determinants of pain in HOA, as well as the significant differences in age, BMI, and KL sum score observed between the MetS+ and MetS− groups [19,25].

The rationale for using HAD in a separate model was to appreciate its proper effect on the association between clinical outcomes and MetS.

Results were presented as odds ratios with 95 ​% confidence intervals (CI) for the unadjusted and adjusted models. The events were the scores above the median (i.e., “high level”). The reference group was “no MetS” (MetS-). The model fit was evaluated using the Hosmer & Lemeshow test.

Subsequently, we explored the association between these patient-reported outcomes and each component of the MetS using logistic regression models. We performed unadjusted analysis of each component of the MetS and two adjusted analysis including all components of the MetS:

- Model 3: adjusted for model 1 ​+ ​Elevated waist circumference ​+ ​Elevated triglycerides ​+ ​Reduced HDL cholesterol ​+ ​Hypertension ​+ ​Elevated fasting blood glucose
- Model 4: model 3 ​+ ​HAD scale.

This supplementary investigation aimed to elucidate potential links between individual MetS components and specific aspects of HOA-related pain. By examining these associations, we sought to gain a deeper understanding of the underlying mechanisms driving pain in the context of MetS.

As sensitivity analyses, AIMS2 pain score and the number of painful joints on pressure were analyzed as continuous outcomes.

- AIMS2 pain score was analyzed using linear regression (normality visually assessed), with results presented as regression parameters and their 95 ​% confidence intervals.
- The number of painful joints on pressure was analyzed as count data using a quasi-Poisson regression model due to overdispersion, with results presented as rate ratios and their 95 ​% confidence intervals.

As with the logistic regression analysis, one unadjusted and two adjusted analyses were performed for each model.

Considering the putative driving role of adiposity within the MetS to drive a potential association between MetS and BMI, we also investigated whether the effect of a 5-point increase in BMI is associated with each outcome criterion using unadjusted logistic regression models.

The significance threshold was set at *p* ​< ​0.05 for statistical analysis. Statistical analysis was performed using SAS v9.4 software.

## 3 Results

### 3.1 Patient characteristics

Among the 426 patients included in the DIGICOD cohort, 352 (82.6 ​%) were included in this study. 74 patients were excluded due to missing MetS data (n ​= ​20) or incomplete outcome/adjustment factor information (n ​= ​54) (figure 1, supplementary file). Of the analyzed cohort, 125 (35.5 ​%) patients were classified as having MetS.

Table 1 outlines the characteristics of HOA patients with and without MetS (MetS+ and MetS). As expected, significant differences were observed in MetS components, and obviously also on BMI (27.1 ​± ​4.5 and 24.0 ​± ​3.8 ​kg/m^2^, p ​< ​0.0001), age at inclusion (67.9 ​± ​7.2 and 65.6 ​± ​7.3, p ​= ​0.01), KL sum score (0–128) (48.6 ​± ​17.6 and 44.8 ​± ​17.4, p ​= ​0.05), and HAD scale (0-21) (13.8 ​± ​6.7 and 12.0 ​± ​5.7, p ​= ​0.01). Table 2 describes the symptoms intensity in the entire population as well as according to the presence or absence of MetS. For each PROMs, the median value was used to define high or low level of symptoms. To search for an association with MetS, each outcome was binarized according to it's median value to define “high symptom” and “low symptom”.

**Table 2:** Description of the evaluation criteria on the study population (n ​= ​352).

Variable	Total population N ​= ​352		Metabolic syndrome N ​= ​125		No metabolic syndrome N ​= ​227	
	P50 [P25; P75]					
	**N**^a^		**n**^a^		**n**^a^	
AUSCAN pain subscore (0–100)	352	20.4 [8.4; 38.1]	125	27.4 [14.0; 43.2]	227	17.4 [7.0; 36.8]
AIMS2 pain (0–100)	352	33.3 [16.7; 41.7]	125	33.3 [25.0; 50.0]	227	25.0 [16.7; 41.7]
VAS hand pain at rest (0–100)	352	15.0 [0.0; 35.0]	125	18.0 [5.0; 36.0]	227	11.0 [0.0; 34.0]
VAS hand pain during activity (0–100)	352	44.5 [22.0; 66.0]	125	51.0 [30.0; 69.0]	227	40.0 [20.0; 65.0]
Number of joints painful on pressure (0-30)	352	3.0 [2.0; 6.0]	125	4.0 [2.0; 8.0]	227	3.0 [1.0; 5.0]

AUSCAN: Australian/Canadian Hand Osteoarthritis Index.

AIMS2: Arthritis Impact Measurement Scales 2.

VAS: Visual Analogic Scale.

a Number of data available.

### 3.2 Association between MetS and pain outcomes

In the unadjusted analyses, the presence of MetS was significantly associated with a higher risk of having elevated pain scores based on various outcome measures AUSCAN pain subscore as primary outcome and AIMS2 pain score, VAS hand pain during activity in secondary outcomes (Table 3).

**Table 3:** Association between metabolic syndrome and pain in hand osteoarthritis according to different assessments^a^.

	Unadjusted model		Adjustment on age, sex, KL total sum score (Model 1)		Adjustment on age, sex, KL total sum score and HAD (Model 2)	
	OR (95 ​% CI)	p-value	OR (95 ​% CI)	p-value	OR (95 ​% CI)	p-value
AUSCAN pain subscore ≥19.8	1.71 (1.10; 2.67)	0.02	1.66 (1.05; 2.62)	0.03	1.56 (0.98; 2.48)	0.06
AIMS 2 pain score ≥33.3	2.03 (1.29; 3.19)	0.002	2.06 (1.29; 3.29)	0.003	1.85 (1.14; 2.99)	0.01
VAS hand pain at rest ≥16	1.32 (0.85; 2.05)	0.2	1.31 (0.83; 2.05)	0.24	1.20 (0.76; 1.89)	0.44
VAS hand pain during activity ≥45	1.78 (1.14; 2.77)	0.01	1.78 (1.13; 2.80)	0.01	1.61 (1.02; 2.57)	0.04
Number of painful joints on pressure ≥3	1.37 (0.87; 2.16)	0.17	1.39 (0.87; 2.22)	0.17	1.32 (0.82; 2.13)	0.25

AUSCAN: Australian/Canadian Hand Osteoarthritis Index.

AIMS2: Arthritis Impact Measurement Scales 2.

VAS: Visual Analogic Scale.

KL: Kellgren-Lawrence.

HAD: Hospital Anxiety and Depression scale.

a Data are OR (95 ​% CI) given for Mets ​+ ​while Mets-serves as reference group.

In model 1, association persisted for these 3 criteria, on the AUSCAN pain score (OR ​= ​1.66, 95 ​% CI (1.05; 2.65), p ​= ​0.03). Furthermore, MetS continued to demonstrate a significant association with an increased likelihood of experiencing elevated pain scores on both the AIMS2 pain subscore (OR ​= ​2.06, 95 ​% CI (1.29; 3.29), p ​= ​0.003) and the VAS hand pain during activity (OR ​= ​1.78, 95 ​% CI (1.13; 2.80), p ​= ​0.01).

In fully adjusted model 2, the statistical association with MetS remains significant for the AIMS2 pain subscore (OR ​= ​1.85, 95 ​% CI (1.14; 2.99), p ​= ​0.01) and for the VAS hand pain during activity (OR ​= ​1.61, 95 ​% CI (1.02; 2.57), p ​= ​0.04). The association between MetS and AUSCAN pain did not persist after adjustment for HAD, although a trend was observed (OR ​= ​1.56, 95 ​% CI (0.98; 2.48), p ​= ​0.06).

Conversely, no significant association was found with pain on VAS hand pain at rest or the number of painful joints under pressure in any analysis.

### 3.3 Association between MetS components or BMI and clinical outcome

We subsequently investigated the relationship between BMI and each outcome considering a 5-point increase in BMI: no significant association between BMI and the outcome measures was observed, as depicted in Table 4.

**Table 4:** Effect of 5-point increase in BMI on each outcome criterion.

Criteria	Effect of 5-points increase BMI on each outcome criterion		
	N	OR (CI95 ​%)	p-value
AUSCAN pain subscore ≥19.8	347	1.21 (0.94; 1.55)	0.14
AIMS 2 pain score ≥33.3	347	1.12 (0.87; 1.44)	0.37
VAS hand pain at rest ≥16	347	1.20 (0.93; 1.54)	0.16
VAS hand pain during activity ≥45	347	1.20 (0.94; 1.55)	0.15
Number of painful joints on pressure ≥3	347	0.89 (0.69; 1.14)	0.37

BMI: Body Mass Index.

AUSCAN: Australian/Canadian Hand Osteoarthritis Index.

AIMS2: Arthritis Impact Measurement Scales 2.

VAS: Visual Analogic Scale.

### 3.4 Exploratory analyses on the association between patients reported outcomes and MetS components

After full adjustment (model 4), we found no significant associations between individual MetS components and the outcomes assessed, except for elevated triglycerides, which were significantly associated with greater pain intensity as measured by the AIMS2 pain subscore (OR ​= ​2.58, 95 ​% CI [1.09; 6.07], p ​= ​0.03). (Tables 4–8, supplemental files).

## 4 Discussion

Considering the role of low-grade inflammation in OA and its symptoms, the present study aimed to investigate the association between MetS and pain in patients with HOA. In this cross-sectional study of patients with HOA, MetS has emerged as a significant factor associated with elevated levels of hand pain, as indicated by various outcomes, independently of structural lesions and psychological factors such as anxiety and depression. Moreover, our findings suggest that MetS not only exacerbates hand pain but also increases the risk of experiencing osteoarthritic pain beyond the hands, highlighting the broader impact of MetS on joint pain. In addition, the association between MetS and symptoms does not appear to be due to BMI, as it did not correlate with a higher risk of elevated OA symptom scores. Finally, MetS as a whole is important, because when we analyzed MetS items separately, we did not observe any relevant association with pain.

OA manifests as a multifaceted condition influenced by various risk factors. A distinct phenotype, termed metabolic syndrome-associated OA, has emerged, showing a higher prevalence in obese individuals with cardiometabolic disturbances compared to their "healthy" obese counterparts [26]. Previous research has suggested a potential link between MetS and increased risk of osteoarthritic pain, particularly in weight-bearing joints [27]. However, there are contradictory conclusions regarding the association between MetS and knee OA because certain adjustments such as BMI were not taken into account in all the analyses, which means that the role of excessive weight on joint pain cannot be ruled out [[28], [29], [30], [31]]. To investigate the systemic role of MetS in OA-related pain with avoidance of the mechanical impact of BMI on pain, studying hand OA appears to be particularly relevant.

In our study, we have found that MetS was associated with hand pain and pain in general in patients with HOA, thanks to the use of several types of PROMS. Importantly, in our study we adjusted on radiographic structural damages and anxiety depression which may influence pain experience in general. Moreover, since the direct role of BMI is discussed in the pathogenesis of OA-related pain as the mediator of MetS-pain relationship, we checked that BMI did not significantly impact the symptom outcomes.

The role of metainflammation and adipose tissue dysfunction in OA-related pain has been recently emphasized using several biomarkers such as adipokines or C-reactive protein (CRP) [32]. In HOA, a pilot study found a correlation between leptin serum levels and the severity of chronic HOA pain [33]. Furthermore, Haugen et al. have shown that patients with HOA experience more severe pain in their hands, feet, and knees/hips with higher BMI; and also than leptin may have a stronger influence on hand pain compared to lower extremity pain; additionally, low-grade inflammation, measured by C-Reactive Protein High-Sensitivity (hs-CRP), might contribute to overall pain in overweight or obese individuals [19]. Overweight or obese individuals often exhibit low-grade inflammation, as indicated by elevated CRP levels, which may contribute to heightened pain sensitivity [18,32]. Recent studies have explored the relationship between biomarkers of adipose tissue dysfunction and osteoarthritis-related pain, revealing associations with pain intensity, CRP levels, and structural joint damage. Unfortunately, we do not have serum adipokines assessment in the DIGICOD cohort to address the role of adipokines in the relationship between MetS and pain.

In our study, we demonstrated that each component of MetS, when considered independently, is not significantly associated with hand pain in HOA. This lack of association may be partially explained by collinearity between the metabolic components, which limits the ability to isolate their individual effects. Conversely, it is the combination of these interrelated metabolic disturbances—captured by MetS as a whole—that is relevant to the observed effect on pain in OA. While literature suggests a link between diabetes and increased hand pain in erosive HOA [34], there is limited current evidence showing a direct association between MetS components and OA-related pain.

As pain is a multifaceted and subjective experience, our aim was to explore its relationship with MetS by employing diverse evaluation methods to strengthen the robustness of our analyses. We utilized both the AUSCAN and VAS pain scales, centered on the hands yet assessing pain at varying intensities (ie, decontextualized for VAS and contextualized for AUSCAN). Additionally, we incorporated the AIMS2 pain score, which permit to highlight a link between MetS and pain in OA without specifying localization. However, we acknowledge that this PROM cannot discriminate between diffuse OA and widespread pain. This is thus reminiscent with previous studies demonstrating a correlation between fibromyalgia and MetS [35,36]. Therefore, it would be advantageous to introduce the pressure pain threshold test (Quantitative Sensory Testing into our analyses, a non-invasive exploration method allowing the investigation of nociplastic pain, as has been done in other studies [19].

Despite the strengths of our study, including a well-characterized cohort and validated MetS definition, several limitations warrant consideration. The cross-sectional may suggest association but not causation since we need for that. However, this study is unique as it is the first to specifically explore the association between MetS and pain in HOA, addressing the systematic link between MetS and digital osteoarthritis pain with a particular focus on the hands.

We used a large cohort of HOA with accurate phenotypes and several PROMs. Additionally, since DIGICOD is a based-hospital cohort, a recruitment bias cannot be avoided and thus generalization to all people with HOA. Our results need to be confirmed in another cohort for external validation, ideally in a family care cohort or in the general population. We admit as a limitation that our approach of including only patients with complete data may introduce bias by excluding individuals with missing variables. While this strategy ensures consistency across analyses, it may limit the generalizability of our findings and does not address the potential biases that multiple imputation could help mitigate. We acknowledge the limitations of dichotomizing outcomes using the median; however, this approach was chosen due to the non-normal distribution of many outcome variables, even after attempted transformations, and to ensure consistency in the presentation of results. Notably, sensitivity analyses of the AIMS2 Pain Score and the number of painful joints under pressure, analyzed as continuous outcomes, yielded results comparable to those obtained using the median (see Supplementary File).

Additionally, using medians as cut-offs, such as the median VAS pain score during activity (45/100), is particularly relevant as it closely aligns with the clinically meaningful threshold of 40/100 commonly employed in clinical trials and corresponding to the PASS criteria [37]. Finally, because BMI of our patients is not notably high in this cohort, it would be valuable to conduct a study within a population of patients with HOA with a higher BMI, possibly from another population.

In conclusion, our study suggests that MetS is associated with both hand and generalized OA-related pain among patients with HOA, in the DIGICOD cohort. These results underscore the complex interaction between metabolic health and OA-related pain and highlight potential pathways to be explored further to understand and manage OA-related pain. Further research is needed to elucidate the underlying mechanisms driving this association and to develop personalized management strategies for affected individuals.

## Ethics

The DIGICOD study complies with the Declaration of Helsinki and obtained all the regulatory and ethics validation from the local regulatory committee (Comité de Protection des Personnes, Paris Île-de-France IV).

## Data availability

Data sharing will be subject to the terms of DIGICOD cohort and the AP-HP data-sharing agreement to ensure all users of the data adhere to the legal requirements of using personal data.

## Authors’ contribution

Alix Charton, Romane Lacoste-Badie, Sophie Tuffet, Alexandra Rousseau, Emmanuel Maheu, Bruno Fautrel, Maxime Dougados, Francis Berenbaum, Alice Courties, Jérémie Sellam participated in the conception and design of the study as well as the interpretation of the data.

Sophie Tuffet, Alexandra Rousseau, Romane Lacoste-Badie carried out the statistical analyzes.

Alix Charton drafted the article.

All co-authors revised it critically for important intellectual content.

Jérémie Sellam gave final approval of the version to be submitted.

## Role of the funding source

DIGICOD cohort is labeled and granted by the French Society of Rheumatology. DIGICOD cohort was supported by an unrestricted grant from TRB Chemedica, who did not take part in the study design, collection, analysis, and interpretation of data, writing of the report or the decision to submit the article for publication.

## Declaration of competing interest

Alix CHARTON received an institutional grant from French Society of Rheumatology.

Jérémie SELLAM reports personal fees from MSD, Pfizer, Abbvie, Fresenius Kabi, BMS, Roche, Chugai, Sandoz, Lilly, Novartis, Galapagos, AstraZeneca, UCB, Grunenthal and Janssen and research grants from Pfizer (ADVANCE 2020 grant), Schwa Medico, and BMS. JS received an academic grant for Going Inside Osteoarthritis-Related Pain Phenotyping network from ERA-NET NEURON.

Francis BERENBAUM received an institutional grant from TRB Chemedica and Pfizer and consulting fees from AstraZeneca, Boehringer Ingelheim, Bone Therapeutics, Cellprothera, Galapagos, Gilead, Grunenthal, GSK, Lilly, MerckSerono, MSD, Nordic Bioscience, Novartis, Pfizer, Roche, Sandoz, Sanofi, Servier, UCB, Peptinov, 4P Pharma, and 4Moving Biotech.

Bruno FAUTREL has received research grants from AbbVie, Lilly, MSD and Pfizer, and consultancy fees from AbbVie, Amgen, Biogen, BMS, Celltrion, Chugai, Fresenius Kabi, Galapagos, Janssen, Lilly, Medac, MSD, NORDIC Pharma, Novartis, OWKIN, Pfizer, Roche, Sandoz, Sanofi-Genzyme, SOBI, UCB, Viatris.

Alice COURTIES has received research grants from Pfizer, Roche, Abbvie, UCB, BMS, Novartis, Janssen, Biogen.

Emmanuel MAHEU declare punctual advice or intervention in symposia for: Expanscience, FIDIA, IBSA, Pierre Fabre, SUBLIMED, TRB Chemedica.

Romane LACOSTE-BADIE, Sophie TUFFET, Alexandra ROUSSEAU and Maxime DOUGADOS don't declare any conflict of interest.

During the preparation of this work the author used ChatGPT in order to correct English, spelling, syntax and grammar. After using this tool/service, the author reviewed and edited the content as needed and takes full responsibility for the content of the publication.
