# Supplementary material for: Metabolic syndrome is associated with more pain in hand osteoarthritis: Results from the DIGICOD cohort
Source: Osteoarthr Cartil Open. 2025 Feb 5;7(1):100573. doi: 10.1016/j.ocarto.2025.100573 (PMC11869907; doi:10.1016/j.ocarto.2025.100573)
Supplement: Multimedia component 1 [file mmc1.docx]

***Supplementary file***

Table 1. Definition of metabolic syndrome according to Adult Treatment Panel III criteria:

| Metabolic syndrome defined by: |
| --- |
| At least three of the following criteria:   1. Elevated waist circumference (≥102 cm in men, ≥88 cm in women), 2. Elevated triglycerides (≥1.5 g/L or drug treatment for high triglycerides) 3. Reduced HDL cholesterol (<0.4 mg/dL in men, <0.5 mg/L in women or drug treatment for reduced HDL cholesterol), 4. Hypertension (systolic blood pressure ≥130 mm Hg, diastolic blood pressure ≥85 mm Hg or antihypertensive medication), 5. Elevated fasting blood glucose (≥5.6 mmol/L or blood glucose lowering medication). |

Figure 1. Flowchart


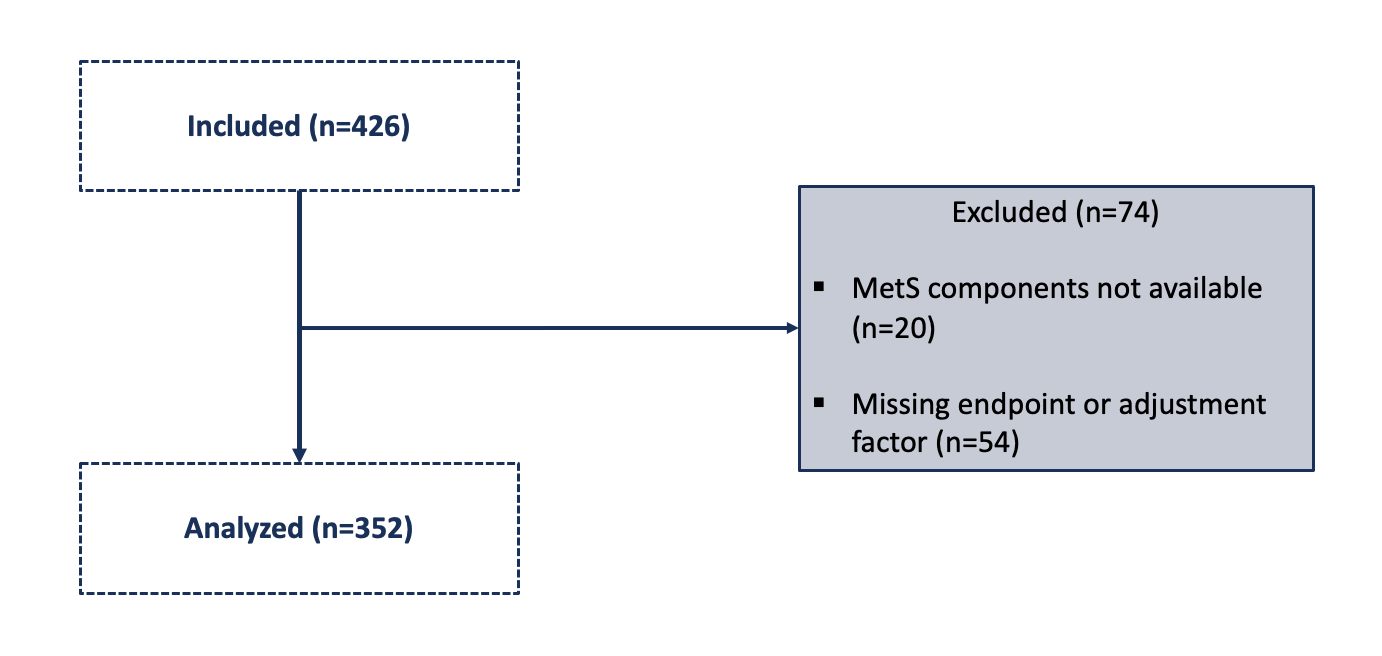


***Sensitivity Analyses of AIMS2 Pain Score and Number of Painful Joints on Pressure as Continuous Outcomes***

This sensitivity analysis revealed a strong association between metabolic syndrome and AIMS2 pain scores (OR=8.86, 95% CI (4.45;13.27, p < 0.0001)) in the unadjusted model, which remained significant after adjusting for age, sex, and KL score (OR=8.63, 95% CI(4.23–13.03), p = 0.0001)), and was still significant after further adjustment for the HAD score ((OR=6.55, 95% CI(2.31–10.79), p = 0.003) (**Table 2**).

Concerning the association between metabolic syndrome and the number of painful joints on pressure, we found in unadjusted model a significant association OR=1.40 (95% CI (1.14;1.71), p = 0.001). After adjusting, in model 1 et 2, association was still significant OR=1.37 (95% CI(1.12;1.66), p = 0.002) for model 1 and OR=1.27 (95% CI(1.05;1.53), p = 0.012)) for model 2 (**Table 3**).

Table 2. Association Between Metabolic Syndrome and AIMS 2 Pain: Results of Regression Analysis

*AIMS2 pain score was analyzed using linear regression (normality visually assessed), with results presented as regression parameters and their 95% confidence intervals.*

| Variable | Non adjusted model | | Model adjusted for age, sex, Kellgren-Lawrence score | | Model adjusted for age, sex, Kellgren-Lawrence score, HAD score | |
| --- | --- | --- | --- | --- | --- | --- |
|  | OR (CI95%) | p-value* | OR (CI95%) | p-value* | OR (CI95%) | p-value* |
| AIMS2 PAIN | 8.86 (4.45; 13.27) | <0.0001 | 8.63 (4.23; 13.03) | 0.0001 | 6.55 (2.31 ; 10.79) | 0.003 |

*Data are OR (95% CI) given for Mets+ while Mets- serves as reference group.

*Test t

Table 3. Association Between Metabolic Syndrome and number of Painful Hand Joints on Pressure: Results of Regression Analysis.

*The number of painful joints on pressure was analyzed as count data using a quasi-Poisson regression model due to overdispersion, with results presented as rate ratios and their 95% confidence intervals.*

| Variable | Non adjusted model | | Model adjusted for age, sex, Kellgren-Lawrence score | | Model adjusted for age, sex, Kellgren-Lawrence score, HAD score | |
| --- | --- | --- | --- | --- | --- | --- |
|  | OR (CI95%) | p-value* | OR (CI95%) | p-value* | OR (CI95%) | p-value* |
| Number of painful joints ON PRESSURE | 1.40 (1.14; 1.71) | 0.001 | 1.37 (1.12 ; 1.66) | 0.002 | 1.27 (1.05 ; 1.53) | 0.012 |

*Data are OR (95% CI) given for Mets+ while Mets- serves as reference group.

*Wald test

AIMS2: Arthritis Impact Measurement Scales 2

Table 4. Results from models assessing the association between the metabolic syndrome components and AUSCAN Pain score ≥ 19.8 (n=352).

| Variable | Non adjusted model | | Model 3 | | Model 4 | | |
| --- | --- | --- | --- | --- | --- | --- | --- |
|  | **OR (CI95%)** | **p-value*** | **OR (CI95%)** | **p-value*** | | **OR (CI95%)** | **p-value*** |
| Elevated waist circumference | 1.71 (1.07 ; 2.73) | 0.03 | 1.44 (0.87 ; 2.39) | 0.16 | | 1.36 (0.81 ; 2.27) | 0.24 |
| Elevated triglycerides | 1.52 (0.98 ; 2.35) | 0.06 | 0.88 (0.40 ; 1.95) | 0.75 | | 0.82 (0.37 ; 1.83) | 0.62 |
| Reduced HDL cholesterol | 1.72 (1.10 ; 2.69) | 0.02 | 1.66 (0.75 ; 3.71) | 0.21 | | 1.77 (0.78 ; 3.99) | 0.17 |
| Hypertension | 1.11 (0.68 ; 1.81) | 0.67 | 1.00 (0.59 ; 1.68) | 0.99 | | 1.01 (0.60 ; 1.71) | 0.97 |
| Elevated fasting blood glucose | 1.22 (0.75 ; 1.99) | 0.42 | 1.19 (0.70 ; 2.04) | 0.52 | | 1.12 (0.65 ; 1.93) | 0.69 |

*Wald Test

Model 3: adjusted for age, sex and Kellgren-Lawrence score

Model 4: adjusted for age, sex, Kellgren-Lawrence score and Hospital Anxiety and Depression scale

Table 5. Results from models assessing the association between the metabolic syndrome components and AIMS2 Pain score ≥ 33.3 (n=352).

| Variable | Non adjusted model | | Model 3 | | Model 4 | |
| --- | --- | --- | --- | --- | --- | --- |
|  | **OR (CI95%)** | **p-value*** | **OR (CI95%)** | **p-value*** | **OR (CI95%)** | **p-value*** |
| Elevated waist circumference | 1.72 (1.07 ; 2.77) | 0.03 | 1.26 (0.75 ; 2.11) | 0.39 | 1.11 (0.65 ; 1.89) | 0.71 |
| Elevated triglycerides | 2.27 (1.45 ; 3.56) | 0.0003 | 2.81 (1.22 ; 6.44) | 0.01 | 2.58 (1.09 ; 6.07) | 0.03 |
| Reduced HDL cholesterol | 1.84 (1.17 ; 2.90) | 0.01 | 0.77 (0.33 ; 1.78) | 0.54 | 0.84 (0.35 ; 1.99) | 0.69 |
| Hypertension | 0.78 (0.48 ; 1.28) | 0.33 | 0.68 (0.40 ; 1.17) | 0.16 | 0.70 (0.40 ; 1.21) | 0.20 |
| Elevated fasting blood glucose | 1.12 (0.69 ; 1.82) | 0.65 | 1.10 (0.64 ; 1.91) | 0.72 | 0.97 (0.55 ; 1.71) | 0.92 |

*Wald Test

Model 3: adjusted for age, sex and Kellgren-Lawrence score

Model 4: adjusted for age, sex, Kellgren-Lawrence score and Hospital Anxiety and Depression scale

Table 6. Results from models assessing the association between the metabolic syndrome components and VAS hand pain at rest ≥ 16 (n=352).

| Variable | Non adjusted model | | Model 3 | | Model 4 | |
| --- | --- | --- | --- | --- | --- | --- |
|  | **OR (CI95%)** | **p-value*** | **OR (CI95%)** | **p-value*** | **OR (CI95%)** | **p-value*** |
| Elevated waist circumference | 1.54 (0.97 ; 2.44) | 0.07 | 1.30 (0.79 ; 2.15) | 0.30 | 1.21 (0.72 ; 2.01) | 0.47 |
| Elevated triglycerides | 1.32 (0.86 ; 2.04) | 0.21 | 0.72 (0.32 ; 1.61) | 0.42 | 0.65 (0.29 ; 1.48) | 0.31 |
| Reduced HDL cholesterol | 1.54 (0.99 ; 2.39) | 0.06 | 1.80 (0.81 ; 4.03) | 0.15 | 1.95 (0.86 ; 4.44) | 0.11 |
| Hypertension | 1.07 (0.65 ; 1.74) | 0.79 | 1.01 (0.60 ; 1.71) | 0.96 | 1.03 (0.61 ; 1.75) | 0.91 |
| Elevated fasting blood glucose | 1.53 (0.94 ; 2.50) | 0.09 | 1.57 (0.92 ; 2.68) | 0.10 | 1.45 (0.84 ; 2.49) | 0.18 |

*Wald Test

Model 3: adjusted for age, sex and Kellgren-Lawrence score

Model 4: adjusted for age, sex, Kellgren-Lawrence score and Hospital Anxiety and Depression scale

Table 7. Results from models assessing the association between the metabolic syndrome components and VAS Pain hand during activity ≥ 45 (n=352).

| Variable | Non adjusted model | | Model 3 | | Model 4 | |
| --- | --- | --- | --- | --- | --- | --- |
|  | **OR (CI95%)** | **p-value*** | **OR (CI95%)** | **p-value*** | **OR (CI95%)** | **p-value*** |
| Elevated waist circumference | 1.56 (0.98 ; 2.48) | 0.06 | 1.38 (0.84 ; 2.28) | 0.20 | 1.26 (0.75 ; 2.09) | 0.38 |
| Elevated triglycerides | 1.44 (0.93 ; 2.22) | 0.10 | 1.16 (0.53 ; 2.51) | 0.71 | 1.03 (0.46 ; 2.27) | 0.95 |
| Reduced HDL cholesterol | 1.46 (0.94 ; 2.26) | 0.09 | 1.22 (0.56 ; 2.66) | 0.62 | 1.35 (0.61 ; 3.01) | 0.46 |
| Hypertension | 0.91 (0.56 ; 1.48) | 0.71 | 0.86 (0.51 ; 1.45) | 0.58 | 0.88 (0.52 ; 1.49) | 0.64 |
| Elevated fasting blood glucose | 1.03 (0.64 ; 1.67) | 0.90 | 0.96 (0.57 ; 1.63) | 0.88 | 0.85 (0.50 ; 1.47) | 0.56 |

*Wald Test

Model 3: adjusted for age, sex and Kellgren-Lawrence score

Model 4: adjusted for age, sex, Kellgren-Lawrence score and Hospital Anxiety and Depression scale

Table 8. Results from models assessing the association between the metabolic syndrome components and number of painful hand joints under pressure ≥ 3 (n=352).

| Variable | Non adjusted model | | Model 3 | | Model 4 | |
| --- | --- | --- | --- | --- | --- | --- |
|  | **OR (CI95%)** | **p-value*** | **OR (CI95%)** | **p-value*** | **OR (CI95%)** | **p-value*** |
| Elevated waist circumference | 1.07 (0.66 ; 1.72) | 0.79 | 0.93 (0.55 ; 1.57) | 0.80 | 0.88 (0.52 ; 1.49) | 0.63 |
| Elevated triglycerides | 1.36 (0.87 ; 2.14) | 0.17 | 0.87 (0.39 ; 1.95) | 0.73 | 0.80 (0.35 ; 1.82) | 0.60 |
| Reduced HDL cholesterol | 1.64 (1.03 ; 2.61) | 0.04 | 2.03 (0.89 ; 4.61) | 0.09 | 2.19 (0.95 ; 5.07) | 0.07 |
| Hypertension | 0.96 (0.58 ; 1.59) | 0.88 | 0.97 (0.56 ; 1.66) | 0.90 | 0.98 (0.57 ; 1.69) | 0.95 |
| Elevated fasting blood glucose | 0.70 (0.43 ; 1.14) | 0.15 | 0.68 (0.40 ; 1.17) | 0.17 | 0.63 (0.37 ; 1.10) | 0.10 |

*Wald Test

Model 3: adjusted for age, sex and Kellgren-Lawrence score

Model 4: adjusted for age, sex, Kellgren-Lawrence score and Hospital Anxiety and Depression scale
